# What’s Right and Wrong in Preclinical Science: A Matter of Principled Investigation

**DOI:** 10.3389/fnbeh.2022.805661

**Published:** 2022-03-09

**Authors:** Laura N. Smith

**Affiliations:** ^1^Department of Neuroscience and Experimental Therapeutics, Texas A&M University Health Science Center, Bryan, TX, United States; ^2^Texas A&M Institute for Neuroscience, Texas A&M University, College Station, TX, United States

**Keywords:** scientific integrity, positive bias, translational neuroscience, power analysis, academic success

## Abstract

The discovery of efficacious treatment options for neuropsychiatric conditions is a process that remains in jeopardy. Contributing to the failure of clinical trials, a strong positive bias exists in the reported results of preclinical studies, including in the field of neuroscience. However, despite clear recognition of major factors that lead to bias, efforts to address them have not made much meaningful change, receiving inadequate attention from the scientific community. In truth, little real-world value is currently attached to efforts made to oppose positive bias, and instead—partially driven by competitive conditions—the opposite has become true. Since pressures throughout our system of scientific discovery, particularly those tied to definitions of individual success, hold these damaging practices firmly in place, we urgently need to make changes to the system itself. Such a transformation should include a pivot away from explicit or tacit requirements for statistical significance and clean narratives, particularly in publishing, and should promote *a priori* power calculations as the determinant of final sample size. These systemic changes must be reinforced and upheld in responsible decisions made by individual scientists concerning the planning, analysis, and presentation of their own research.

## Forward

The ability of preclinical studies to effectively inform human clinical trials, particularly for neuropsychiatric conditions, has been increasingly called into question with greater and greater fervor. Preclinical work, conducted using non-human animals or tissue culture (animal or human), generally falls under the category of basic science and provides the foundation for clinical trials (an applied science). In other words, we rely on preclinical work conducted by basic scientists, to guide which costly clinical investigations are most beneficial for successful therapeutic development in humans. However, even when considering relatively impactful preclinical work in neuropsychiatry, only an abysmal 9% of the resulting clinical trials successfully identify an effective treatment—that is, show a statistically significant improvement in symptoms for participants given the treatment compared to those given the placebo. Pharmaceutical companies have consequently been moving away from neuropsychiatric drug development, a betrayal of opinion that should be ringing alarm bells for many. Yet when I look around, I see basic scientists under pressure to produce, who are surviving using the rules of the game as they have been defined for them.

My lab uses mouse models to conduct basic research in the study of neuropsychiatric and neurodevelopmental conditions, including fragile X syndrome—an area of study that has had its own major failure in drug development. Reading over lists of accused deficiencies in preclinical research (e.g., chronically low statistical power, misuse of hypothesis testing), there are real problems in how we collectively collect and report data. I have been aware of such flaws since early in my training, but instead of these problems being increasingly resolved over the course of my career, as I had idealized, I now feel myself an unwitting cog—nearly fully assimilated into the very system holding the flaws in place. There are many contributing factors, but competition, perhaps particularly in neuroscience, has incentivized the use of shortcuts and cultivated a perpetual need for results to be groundbreaking, while disincentivizing intellectual caution and humility. We insist on clean narratives regarding scientific results. We do not value negative findings. We promote the early sharing of data (but are forgetting to emphasize best practices to protect against bias). We are entirely beholden to statistical significance.

These pressures have distilled successful science into something quite unrealistic, resulting in an overwhelming positive bias in the literature that now obscures our ability to discern effective treatments. Worse, we are directly teaching these missteps to the next generation of scientists. If we do not make corrections to our expectations and practices soon, what impact will this have on people affected by neuropsychiatric conditions now and in the future? As a basic scientist, I know us. We are noses *on* the grindstone. We are doing good in our own way. We are clamoring to maintain a spot in the unforgiving machine that we know as science. But I think for all these reasons, we are not meeting the moment.

## The Web in Which We Hang: Putting Flaws in Basic Research Into Context

Problems in the research practices used in both clinical and preclinical studies have contributed to a growing “positive bias” in the published literature. This distortion arises most directly from constraints on publishing, namely a near requirement for statistical significance, which has been described for over 60 years (Sterling, 1959; Rosenthal, 1979) and affects both basic research and clinical trials (Bowcut et al., 2021). In the simplest interpretation of this problem, non-significant results are less often published, being instead exiled to the figurative “file drawer,” a phenomenon clearly widespread across disciplines and countries, but that appears to affect research from the United States more than the United Kingdom (Fanelli, 2012), for example. One consequence is that we, as scientists, do not benefit from knowledge of others’ “negative” experiments, so we waste resources and time performing them again, and as Sterling (1959) illustrated adeptly, “such research being unknown to other investigators may be repeated independently until eventually by chance a significant result occurs—an ‘error of the first kind’—and is published.”

Speaking from the preclinical side, it is critical to examine these flaws of basic research in their natural habitat, amongst the realities of the system we have built for scientific discovery, as products of the pressures that keep them in place. We are fortunate that the ideal of ethical responsibility remains a cornerstone of scientific investigation. However, basic researchers are squeezed by increasing demands—many driven by the role of competition in our scientific process; and thus, in tandem, the system has evolved to reward ethically questionable behavior (Devereaux, 2014; Kretser et al., 2019), pushing more researchers firmly into gray areas. As happens with many things, the standards erode so slowly that it is difficult to identify it as such, in seemingly tiny decisions and personal evaluations made in lab after lab, concerning work that is paramount to each evaluator’s relevance, success, and livelihood (see Table 1, Self-assessment). Often, these tiny decisions may even appear justified, made in the service of solving the world’s problems. Except that collectively, they completely undermine the premises of true discovery. All these problems have been pointed out, some even chipped away at, but the web entangling us is formidable.

**TABLE 1:** Self-assessment and rules to help protect against bias when conducting research with null hypothesis significance testing.

	Self-assessment
**As a scientist, have you ever…?**	□ Omitted confusing or contrary data from a grant application, presentation, or publication? □ Used the results of a null statistical test to suggest boundaries on other significant findings without considering power? □ Based sample size on the number usually needed to achieve significance? □ Analyzed the data to determine whether to add more animals? □ Determined the required sample size by whether the predicted effect is statistically significant? □ Said of nearly significant data, “I think we just need to increase the sample size, and we’ll likely see significance?” □ Decided whether to remove outliers based on results? □ Reported only a significant subset of multiple experiments?

	**Proper conductance of null hypothesis significance testing**

**Rule #1:** **Make a solid plan to test your predictions before conducting the research**	• At the prediction stage, put your scientific question into words. • Plan targeted tests that address that question carefully, and for each, formulate one or more specific and testable hypotheses. • Before proceeding much further, decide which approach to use for Rule #2 and check over any guidelines or requirements. • Write each hypothesis in the same terms that you will later use to discuss your results (e.g., Hypothesis 1: Group A will have significantly more of Specific Dependent Variable X than Group B; Hypothesis 2: Group A will not be significantly different from Group C). These hypotheses should be tied to critical tests of your scientific question. • Record what each hypothesis indicates concerning your scientific question (both if supported and if not), as well as limitations of each test. • For each experiment, estimate the effect size and, using an acceptable power level for your field, run *a priori* power analyses to determine the final sample size for each. • Run the power analysis for your omnibus (overall) planned analysis, as well as for any *post hoc* analyses that would be required (pending omnibus test significance) to test your hypothesis. Use the higher calculated sample size. • Define inclusion and exclusion criteria to be applied before and after data collection, respectively. • Discuss this plan with all others participating in the research.
**Rule #2:** **Write the plan in stone, in public**	• Preferably, submit a Registered Report to a participating journal. • In addition to or *in lieu* of the above, deposit your plan in a well-accepted analysis plan repository, such as the Open Science Framework (https://osf.io). Link to this preregistration in all publications and presentations resulting from the work. • Least preferred (but at minimum), record all aspects of your research plan. Get feedback from investigators not involved in the work, then make a copy of the final plan available to everyone involved in the work.
**Rule #3:** **Run experiments following the plan and using responsible experimental practices**	• Follow your pre-existing research plan. • Strive to equalize group representation across all experimental cohorts. • Apply inclusion and exclusion criteria before running any statistics and limit preliminary analyses of data to that strictly needed. The need to view and share data early renders the plan and *a priori* power calculations especially essential. • Whenever possible, blinded experimenters should remain blind to interim results. • Include the research plan in any related papers and relevant details (such as power analysis results) in presentations of the work, preliminary or otherwise.

## To Understand the Problem, We Must Examine the Goal: How Publishing Ideals Warp Science

To survive, basic scientists must publish routinely. It is a constant point of evaluation, in every funding application and every promotion or tenure review. Critically, publications are the currency on which transactions for scientific success depend—with high profile publications being especially valuable. Numbers and impact of publications increase other markers of success—awarded grants, as well as invitations to speak, collaborate, and review. Falling behind even a little means it can be very difficult, if not impossible, to catch up. Certainly, there must be demarcations of worthiness regarding publishable scientific work, but unfortunately, presence of statistical significance has become a primary filter in this process, and its value is reinforced in almost all aspects of the scholarly enterprise (Dwan et al., 2013). Results lacking statistically significant differences between studied groups, unless in the service of a larger set of significant findings, are assigned very little value at all (save the largely unrecognized goodwill effort it takes to publish them). Further, when “negative data” are published, they are cited less frequently than positive findings (Duyx et al., 2017), particularly in neuroscience and the biological sciences (Fanelli, 2013).

Not only is there little incentive to publish null findings, but it can also be difficult to do so (Stern and Simes, 1997; Decullier et al., 2005; Fanelli, 2010, 2012; Duyx et al., 2017; Scherer et al., 2018). It is sometimes recommended to submit negative data to a journal that has previously published a positive result on the same research question. However, I’ve very rarely (never?) come across this sort of article, making me wonder if it represents a feasible avenue to publication. Special journals for negative data have had names like *The Journal of Negative Results* and *The All Results Journal.* There are laudable goals here, for sure, but under our current definitions of career success, I must ask—are we *trying* to shame scientists? The fact that journals were created especially for null results, places they could be corralled, suggests the level of disdain our system has for them. In a perusal of journals for null findings I found that some are now defunct, while those remaining publish between zero and 12 articles per year. At least one negative data journal announced that its own example started a wave of change amongst journals, and thus, it is no longer needed. Count me dubious.

To be clear, the metric of statistical significance does not align, by nature, with the use of proper scientific methodology or necessarily with promise of scientific talent, and thus, while we regularly use it as such, it is likely a poor indicator of either. One can suggest that increased incidence of statistically significant findings is a natural result of asking well-founded, hypothesis-driven questions, but this premise cannot account for the estimated 6% increase in reported positive findings per year between 1990 and 2007 (Fanelli, 2012). And as pointed out by Boulbes et al. (2018), poor replicability of studies further negates this idea. Instead, the possibility that “well-founded, hypothesis-driven questions” are increasingly based on spurious findings—driven by positive selection bias and biased data analytical practices—and are feeding self-reinforcing, false scientific avenues (Fanelli, 2012), as may be indicated by our clinical trial failures, is a serious concern. At best, the high value of significant findings gives luck an outsized role in determining a scientist’s success—a career that may require 10 or more years of post-baccalaureate training, during which time it is typical to earn limited wages. At worst, it encourages scientific fraud (Fanelli, 2009; Devine et al., 2021). In any case, the Holy Grail of statistical significance serves as a linchpin for common missteps in preclinical research that will be discussed below—because, yes, we are rewarded for making such missteps and punished when we don’t.

## Getting Turned Around: Letting the Pursuit of Career Success Guide Scientific Practices

### Cherry-Picking Our Data

The mantra “follow the data” is repeated regularly in academic environments and, in my experience, is taken seriously, but inevitably when the story is not perfect, scientists can be punished harshly (Editorial, 2020; Hoekstra and Vazire, 2021). Even small indications that data do not wholly align with a grant hypothesis can be the death knell of an application. The nature of revisions requested during manuscript review are also often guided by expectations of the “perfect story,” and publishing null data, as already discussed, can be an uphill battle in many journals. Scientists have heard the requirements for success loud and clear: build a story that our (positive) data entirely support. But in truth, the world is complex; as scientists we are observing narrow slices of the picture, and data often drive a less cohesive story than the one people want to hear. As a result, the mantra “follow the data” gets turned around, and study results that don’t quite fit have a way of getting left out, or if included, may have their import explained away. If we were truly “following the data,” and likewise allowed flexibility when data don’t match a storyline fully—if we could acknowledge (indeed, remember) that there are unknown and unforeseeable parts of scientific discovery—then the practice of scientific storytelling might be less problematic. Instead, having risen to be nearly a tenet of academic success, I think the pressure to tell a story with scientific work may be one of the more dangerous scientific missteps. Our system of **scientific** discovery expects a story, and once we have one, we tend to defend it (I should pause here and acknowledge that while I did set out to build my scientific house with brick, I find it now to be made of glass. Perhaps as the adage says, people living in glass houses should not throw stones—unless they need to remodel.).

### Power and When to End a Study

Erosion of scientific integrity is unfortunately aided by ignorance of correct statistical practices (Motulsky, 2014), the most common of which may be failure to calculate and observe power-based sample size estimations. “Statistical power is the probability that a study yields a statistically significant effect, if there is a true effect to be found” (Lakens, 2015). It indicates the ability to detect a statistically significant outcome (at a given alpha level, such as *p* < 0.05), given a specific sample size (number of participants or animals) and effect size. So, a power of 0.50 means that a true positive effect (correct rejection of the null hypothesis) would be detected 50% of the time if a study were to be repeated using the same conditions, and in neuroscience, a power level between 0.80 and 0.95 is generally considered desirable. Performed prior to a study, or *a priori*, using an estimation of the size of the effect being measured (small, medium, or large) and the desired power level, power analyses determine the required sample number in each group. Power-related problems have been noted repeatedly in neuroscience (Button et al., 2013; Nord et al., 2017), and likely play a role in the dismal success of neuropsychiatric clinical trials. Notably, if there is no *a priori* power-based determinant of the sample size, conditions are primed for determining sufficient sample size along the way, generally after looking at iterations of results as we increase the sample size (i.e., “optional stopping”). Here again, we have gotten turned around, and there is a tendency, I think, to view statistical significance as proof of sufficient power. In fact, low power increases the likelihood of observing statistical significance when there truly is none (Ioannidis, 2005; Button et al., 2013).

Likewise, looking at iterations of the results opens the door (generally after observing near significance) for the “realization” that some portion of samples doesn’t belong or is problematic to include for some reason. If at any time after looking at results, the authors re-form their hypotheses around these observations, it is known as “hypothesizing after results are known” (HARKing), creating hypotheses that were not predicted and thus violating conditions of null hypothesis significance testing (Kerr et al., 1998; Bishop, 2019). There are other problems: trying out several analyses and selecting the significant ones, known as “*p*-value hacking,” distorts findings and can lead to the acceptance of most any outcome (Head et al., 2015; but see, Botella and Suero, 2020). And running multiple tests inflates the likelihood of finding significance. Observing outcomes prematurely increases the likelihood that an unpromising study (i.e., generally having group differences that do not approach *p* < 0.05) will be abandoned before it is sufficiently powered. Worse yet, those underpowered “negative” findings may be reported or used to establish “boundaries” for other putatively significant effects.

Regarding the required sample size for a properly powered study, it is likely larger than you would estimate on your own. Howells et al. (2014) report that most stroke studies use fewer than 10 animals per group, then they go on to make power-based arguments for using > 20, to upward of 50, animals per group. Reading this, I am reminded how a power analysis can leave me shocked and despondent. We need *that* many animals per group?! So often I think, “surely there’s been a mistake.” Particularly as an early career lab, how can we possibly afford to put that much time and money into a single study when expectations for progress and publishing are patently incompatible? And most terrifying—if there were to be no major significant finding from such a study, there would very likely be no career value in return. Despite the critical nature of power analyses for null hypothesis significance testing, I think the phrase I have heard repeated most often in neuroscience is, “we typically need X number of animals per group to see an effect in this test,” which gives the impression that scientists often rely on experience to determine sample size. How could the mode of testing alone effectively determine required sample size when we are examining the relationships amongst different variables with different effect sizes? Was the avowed sample size originally determined by a statistically significant finding? Does anyone ever compare their experience-based estimations to *a priori* power analyses?

### How and When to Share Our Science

As I see it, problems of underpowered and “optional stopping”-based studies are buttressed by the expectation, indeed requirement, that scientists share their unpublished (often unfinished) work as a regular part of the scientific enterprise—at their home institutions, as invited speakers to other institutions, in grants, and at conferences. Conferences overvalue nascent data, likely to ensure their own continuing relevance, as well as to entice attendees with the latest findings. Grant reviewers, for their part, are increasingly looking for certainty in the form of preliminary success. In theory, sharing unpublished science promotes rapid scientific progress—a decent goal; however, to share work, we must be unblinded to our experimental groups, and an iteration of the results revealed and analyzed. Unless you have a robust plan based on prediction (i.e., before you knew any results), these revelations can re-form hypotheses and shape findings. Even with a robust plan, the value of early results is somewhat questionable since they (should) often change. And how many talks have you attended where a discussion of sample size ensued over a graph? Comments I have heard regularly over my career include, “we likely need just a few more animals here to see significance,” and “it seems like your study is possibly underpowered—the effect is almost there.” Here again, we’ve placed the horse before the cart (and for the record, my glass house is now in pieces).

Other disparities in expectation versus reality blur the lines of ethics for scientists trying not to tank their careers. Labs must be ever productive, but in academia, we are to meet this goal while continually bringing up the next generations of scientists—a truly magnificent responsibility that holds a great deal of honor and joy for most of us—but inherent in this process are mistakes. Sometimes big mistakes that can derail months of work. Trainees should be afforded inevitable missteps while learning to conduct science, but the system simply doesn’t acknowledge this reality. Thus, principal investigators sometimes face an agonizing choice: scrap a study in order to uphold scientific integrity or salvage data in less-than-perfect ways to stay “on-track.” Here again, at best, the system favors the lucky and at worst, the dishonest. Further, investigators have ever-increasing amounts of information to keep grasp of and increasingly are subject to the python grip of “shadow work” (Flaherty, 2016; Taggart, 2021). These added tasks, doled out not only by a scientist’s institution, but also by publishers and grantors—along with the relatively new responsibility to build and maintain an online presence—are ratcheting in nature and held in place by the unspoken reality that there is always someone else willing to jump through these 8,394 hoops. But the more hoops, the less time for the attention that solid research requires. In short, there is less and less time and flexibility to take the lengthier, scientifically responsible path without derailing academic success, and thus, it becomes the road less traveled. We should all be asking, is this our best system for producing accurate science?

## Can We Handle the Truth? Motivating “Disinterest” in Our Scientific Inquiries

“What could it hurt for *me* to look at *my* results early?” “Sure, removing outliers after viewing the results is not ideal, but the effect is right there.” “This effect must be real. I’m sure these few problem mice are just from a different population.” I think, as a scientist, it is easy to make a common, faulty assumption: namely that a significant effect is the same as an objective truth to be revealed, no matter what. Viewed this way, an experiment is less an investigation and more whittling a block of wood into your favorite animal. This assumption may help us circumvent the problematic reality, which is that we do (and must, under the circumstances) deeply care about whether our results are statistically significant (Mahoney, 1979). Now, take these seemingly innocuous and tiny decisions—ones that may absolutely be necessary to preserve a career, no less—and multiply them by another fault that very commonly sinks us as humans: we fail to consider our actions on a different scale than the one we directly experience. When you expand your view and simultaneously consider the warnings about positive bias in our literature, it is impossible to “unsee” how we got here. Unfortunately, now that we are here, in this troubled state, motivating individual scientists to simply make the right changes to their scientific practices—when there is no guarantee that any other scientists will also make these changes—will be nearly impossible. By now, what we should know about human nature is that we find ways to rationalize our behavior when our survival or well-being is at stake. Therefore, if the system continues to reward ethically questionable behavior, or to nurture incompatibility between ethically responsible behavior and career success, we all remain vulnerable to these short-sighted faults. And collectively, our choices mean the difference between revealing scientifically determined truths about our natural world and a finely carved pony.

## In Defense of Preclinical Science and Scientists

After being critical, I need to give a defense of preclinical science and scientists. I am not saying that preclinical research is futile, and I want to clearly endorse continued use of animal models in neuropsychiatric basic research. Some issues with animal models not-withstanding, the fact that preclinical work indiscriminately supports ultimately successful, as well as unsuccessful, therapeutics very likely reflects overwhelming positive bias in publishing. At minimum, we should observe the impact of animal studies that are not tainted by this bias before deciding their worth in the field. And while no basic scientist has entirely pure motives—for one, the system we have built does not support that developmental trajectory—I feel strongly that the vast majority are solidly well-intentioned. I see evidence everywhere of scientists trying to uphold foundational ethical standards concerning their work. When I have heard the indiscretions I quote as examples in this article—indeed, when I make these same gaffes—I don’t think it is borne of a malicious effort to succeed at all costs. The problem is much more complicated, part clear-eyed view of scientific pressures, part misunderstanding of the magnitude of costs, accumulating omissions in training, and a collective slippage in acceptable practice.

## Doing Our Part: Changing Our Individual Expectations as Scientists

As scientists, each of us stands continually judged by the scientific system, and we each contribute in many ways to the collective expectations pursued and held in place by the system. At the end of the day, however, academic and granting institutions, as well as publishers, are made up of and rely on individuals, including scientists—we need only to adjust our expectations. First, let’s start with the research conducted in our own labs, where it is critical to hold yourself and other lab members to the proper use of null-hypothesis significance testing. Here I reiterate rules that should be followed to ensure proper conductance of research (Table 1, Proper Conductance of Null Hypothesis Significance Testing), which in whole or part have been outlined by many previous authors (e.g., Murayama et al., 2014; Kiyonaga and Scimeca, 2019; Harris, 2020). Rule Number 1: Make a solid plan to test your predictions before conducting the research. Null hypothesis significance testing relies on prediction, and prediction is only possible before you have observed any results. That is, throughout the study, you should operate on a plan created back when you were naïve of any outcome. The plan must include a power analysis, which can be performed using a statistical software package or the free program called G*Power.^1^ It is critical to use the *a priori* power calculator options according to each of your planned statistical tests, and you should ideally power your study to the level of any *post hoc* tests required to test the hypotheses. The plan should include specific rules regarding the inclusion of participants and exclusion of data, including outliers. Kiyonaga and Scimeca (2019) do an excellent job of anticipating considerations that may arise during the research stage. Seek feedback and modify the plan accordingly, particularly if you will not submit a Registered Report.

Rule Number 2: Write the plan in stone, in public. Do this, preferably, by submitting a Registered Report for journal peer-review,^2^ and/or preregister an analysis plan in an online repository, such as the Open Science Framework.^3^ Rule Number 3: Run experiments following the plan and using responsible experimental practices. Ensure that all groups are represented (as equally as feasible) in any given cohort. Apply inclusion and exclusion criteria before running any statistics and limit preliminary analyses of data to that strictly needed. Ideally the policy would be to complete the entire study based on the *a priori* power analysis before unblinding, compiling, or presenting results; however, these standards are unrealistic. As scientists we must share our findings before they are complete, and when trainees are new to a technique, looking at emerging data may identify mistakes before precious time and resources are wasted. This need to view and share data makes the plan and *a priori* power calculations even more essential, so that hypotheses won’t be changed mid-study, so that final sample size isn’t debatable, and to avoid *post hoc* data-editing, however well-intentioned. Whenever possible, blinded experimenters should remain blind to interim results. Include your research plan in any related papers and relevant details (such as power analysis results) in presentations of the work, preliminary or otherwise.

Ultimately, we scientists must personally drive some of the change that is needed in our system. As reviewers (see Table 2, Reviewers), we can incentivize scientific modesty, for example, by hinging publication on article titles and interpretations that do not overstate findings (Hoekstra and Vazire, 2021; Wagenmakers et al., 2021). We can also request that *a priori* power analyses, hypotheses, and effect sizes be clearly delineated (Harris, 2020). It is difficult, but when we put on our “reviewer hats,” we must stop insisting on a polished story, implicitly or otherwise. Instead of pushing for a self-contained story, try to put findings into a larger context, acknowledging that (a) there are gaps in our knowledge and (b) existing publications are likely overly subject to positive bias. Consider your own work in this same light, and if you encounter reviewers who show discomfort with data that don’t neatly hang together or who promote unforgiving wording or interpretations, kindly point out that these requests are damaging to scientific progress. Take opportunities to lead by example and prioritize scientific modesty when teaching trainees. Resist the temptation to withhold data that don’t fit “the story.” That departmental journal club with a habit of slaughtering every article with any perceived inconsistency in findings? Speak up and gently open the door for the possibility of real uncertainty in science. Do you understand statistical power analyses? Can you explain them to your trainees (or if you are a trainee, to fellow trainees)? If not, make it a priority to start understanding and put your efforts up front when you share your work with other scientists. The goal is that these habits, formed to protect against bias in our work, will become normal to encounter throughout our scientific enterprise. Fortunately, as researchers, we have considerable control over its content.

**TABLE 2:** Suggestions for changes at the “system” level, aimed at providing appropriate incentives for, as well as normalizing, best practices to protect against bias in scientific discovery.

	Systemic changes needed to correct scientific discovery
**Journals/publishers**	*Concerning Registered Reports* • should begin or continue to accept Registered Reports, committing to publish well-planned research regardless of results • should, at the publisher level, make journal lists searchable by availability of a Registered Report submission option *Concerning Unregistered Manuscripts* • should pivot away from explicit or tacit requirements for statistical significance, as well as for cohesive “stories” • should ensure rigorous methodological practices and appropriate statistical analyses, ensuring that all experiments be sufficiently powered • as part of this process, should verify pre-registered power analyses, allowing flexibility for studies during a transition period *Any Submission Type* • different tiers of journals may still consider other factors, including elegance or complexity of study design, as well as how thoroughly scientific questions are investigated, according to their own standards
**Reviewers**	• should stop (implicitly or explicitly) insisting on a clean narrative • likewise, should tolerate findings that are incongruous with other results • should incentivize scientific modesty, for example, by hinging publication on article titles and interpretations that do not overstate findings • should request that *a priori* power analyses, hypotheses, and effect sizes be clearly delineated • should keep requests for additional lines of investigation consistent with the expectations of the journal
**Forums for early reporting of scientific findings**	*Conferences and Societies* • should define a preference for showing only final study sample sizes determined by *a priori* power analysis, but at minimum require preliminary data to be backed by *a priori* power calculations to determine final sample size • should recommend that blinded experimenters remain blind to interim results *Preprint Servers* • should set expectations of robust scientific methods by requiring information about how and when final sample size was determined • should offer a way to link to preregistered analysis plans *All the Above* • should allow flexibility for studies during a transition period, but thereafter increasingly devalue studies without a Registered Report or preregistration
**Institutions and granting bodies**	• should emphasize distinction between stand-alone pilot studies (used to generate hypotheses) and preliminary data • should promote and value publications of null findings • should use care when shifting responsibilities to principal investigators • should strive to offset new responsibilities with the removal of other binding tasks

## Reweaving the Web: Realigning Our Scientific System With Best Practices

Unfortunately, it can be extremely difficult to change human minds and practices once something becomes established. Indeed, if our only tool is raising the issue every time a scientist makes these mistakes—that is, if we fail to revise the system itself to better reward the desired set of principles—science and our society will be worse for it. Likewise, it is not enough to encourage scientists to publish their carefully procured null results and to encourage others to see them as having real value; such results must *have* real value according to the system in which we function. Let us first consider solutions for these problems in publishing (see Table 2, Journals/publishers). Clearly, there must be standards for publication that are relevant to how the work is conducted, at minimum. There also must be reputable journals that follow agreed-upon standards of publishing, or alternatively, a way to certify that such standards have been followed. Otherwise, the legitimacy of any scientific work becomes questionable, and the common shortcuts that we inevitably use to ascertain integrity, dissolve. To do their part, scientific journals should subject all submitted work to the same scrutiny concerning methodology and decouple publishing decisions from statistical significance.

Fortunately, a highly promising path (Chambers and Tzavella, 2021) toward rehabilitating publication integrity of hypothesis-driven research was started almost 10 years ago, when the idea of Registered Reports was applied to basic science and implemented at *Cortex*. Using this special submission format, authors propose a research rationale and experiments, including detailed hypotheses and methods, as well as calculations of final sample sizes, then they receive peer-review feedback and a decision of “In Principle Acceptance” (IPA) or Rejection—all before the experiments are done. A similar registration process exists at some journals for replication studies called Registered Replication Reports. Assuming the proposed methodology is soundly carried out, IPA guarantees that the manuscript will be published, regardless of results. Registered Reports are a golden solution; not only do they help ensure research integrity by limiting the ability of scientists to inadvertently (or knowingly) treat results as predictions, but they also given attention to the development of a solid research design and ensure that both positive and negative results produced by careful science end up in the literature. Best of all, by guaranteeing acceptance for publication, they provide a much-needed safety net for researchers who have made their scientific investigation more rigorous and, as a result, are less likely to benefit from faster science or spurious significance (Stoevenbelt, 2019).

What is unfortunate is that despite the passage of several years, Registered Reports haven’t been widely adopted by journals. As of late January 2022, only one out of 12 journals from my own publication list, over half representing neuroscience, included the Registered Report submission option. In the author guidelines, one additional journal expressed that preregistered analysis plans can be linked in the published manuscript. Least effective, a third journal required that lead authors sign a “Declaration of Transparency” to ensure the manuscript’s contents are not misleading. Importantly, such a signature requirement arises only after weeks/months/years of research has been done. Then, just as the author is seeing the light at the end of a manuscript submission, a little box appears, and if they don’t sign, the whole shebang will be scrapped? A system relying on eleventh hour author declarations, or even optional preregistered analysis plans (which are a significant improvement), lays entirely too much onus on the individual researcher and doesn’t provide incentive for them to do the right thing. Journals need to acknowledge their role in the problem of positive publication bias and commit firmly to better practices (such as, Editorial, 2020). While both preregistration and Registered Reports require many extra steps and better planning on the part of the researcher, Registered Reports critically also require that some responsibility and risk be held by the journal and thus, provide a promising way forward.

Other players in the scientific enterprise need to step up, including those that provide platforms for sharing data that haven’t yet been peer-reviewed and are incomplete or preliminary (see Table 2, Forums for early reporting of scientific findings). Major granting institutions have required a discussion of power analysis for a while, and it is time to integrate that expectation more fully into forums for early scientific reporting. Such standards should be held by organizations that host scientific conferences, as well as by preprint repositories. Indeed, the development of preprint servers is sublime evidence for how rising expectations in our scientific system break across the backs of researchers. Journal reviewer expectations of statistical significance and a “complete” story slow or completely derail publication, while at the same time, pressures from scientists’ home and granting institutions demand more and faster publications. While hastening availability of results and evidence of productivity, preprint servers do little to address the problems that led to their development. Now that they are part of our scientific system, they should also set expectations of robust scientific methods by requiring information about how and when final sample size was determined. Such repositories should also offer a way to include links to preregistered analysis plans. Similarly, conferences should define expectations for presenting unpublished studies that, at minimum, encourage and normalize the systematic reporting of *a priori* power analyses, alongside each result. They should also recommend that blinded experimenters remain blind to interim results and promote preregistered studies. For these changes, flexibility will need to be allowed for studies during a transition period, but following that, the reporting of findings most vulnerable to bias (i.e., those lacking the Registered Report or preregistered analysis plan) should increasingly be discouraged and de-valued.

Granting institutions, for their part, should outline distinctions between stand-alone pilot studies, which can be used to generate hypotheses without introducing bias to a full experiment, and preliminary data generated as part of a yet-to-be-completed study (see Table 2, Institutions and granting bodies). Academic institutions should enhance education surrounding these issues of power, as well as pilot versus preliminary studies, and should incentivize use of Registered Reports and preregistration. Both granting and home institutions should move to value publications reporting negative and positive findings equally, particularly for purposes of determining a scientist’s productivity, which may influence award and tenure decisions. Lastly, home, granting, and publishing institutions should use care when shifting responsibilities to principal investigators and should strive to offset preregistration-related and other new responsibilities with the removal of other binding tasks. Especially during the early stages of adoption of these practices, having an office or personnel dedicated to assisting researchers in this endeavor could be especially effective.

## Conclusion

I know that the changes we need to make to our system of scientific discovery are huge, and many suggested here will seem unrealistic. Some may say, “we always did it this way, and there was never any problem.” Except that there was, it was just out of sight, or it hadn’t yet grown to the current proportions, or they fail to see the connection between their behavior and the detrimental outcome. And yes, we will always be drawn to scientific findings that show statistical group differences, a preference that is unlikely to be erased from our collective conscience. Despite all these worries, what I know is that the system cannot continue to press scientists in these ways and expect their personal integrity to keep winning out—indeed, responsible scientific behavior and success are now increasingly at odds. Nor can we throw up our hands and accept these critical problems in preclinical science as a foregone conclusion. The warning: a system that doesn’t allow a scientist to be successful in the absence of flashy findings, or one that so strains a scientist’s timeline and resources such that conducting responsible science becomes a liability, is a dangerous one. Indeed, it is a reliable way to construct a house, not of brick or even glass, but of cards.

## Data Availability Statement

The original contributions presented in the study are included in the article/supplementary material, further inquiries can be directed to the corresponding author.

## Author Contributions

LS wrote the manuscript.

## Conflict of Interest

The author declares that the research was conducted in the absence of any commercial or financial relationships that could be construed as a potential conflict of interest.

## Publisher’s Note

All claims expressed in this article are solely those of the authors and do not necessarily represent those of their affiliated organizations, or those of the publisher, the editors and the reviewers. Any product that may be evaluated in this article, or claim that may be made by its manufacturer, is not guaranteed or endorsed by the publisher.

## Footnotes

1
https://stats.oarc.ucla.edu/other/gpower/

2
https://www.cos.io/initiatives/registered-reports

3
https://osf.io/
